# Signals of local bioclimate-driven ecomorphological changes in wild birds

**DOI:** 10.1038/s41598-022-20041-w

**Published:** 2022-09-27

**Authors:** Mylswamy Mahendiran, Mylswamy Parthiban, Parappurath Abdul Azeez

**Affiliations:** 1 Sálim Ali Centre for Ornithology and Natural History, Anaikatty, Coimbatore, Tamil Nadu 641 108 India; 2 Agricultural College and Research Institute, Tamil Nadu Agricultural University, Coimbatore, Tamil Nadu 641 003 India; 3 Post Graduate & Research Department of Computer Science, Government Arts College (Autonomous), Affiliated to Bharathiar University, Coimbatore, Tamil Nadu 641 018 India; 4 Department of Environmental Science and Management, Bharathidasan University, Tiruchirappalli, Tamil Nadu 620 024 India

**Keywords:** Image processing, Climate-change ecology, Sexual selection

## Abstract

Across disciplines—biological, ecological, evolutionary, or environmental—researchers increasingly recognize the importance and the need for cost-effective, non-invasive techniques for in-situ morphological measurements of organisms in diverse research contexts. By applying a non-invasive technique using digital images taken under field conditions, we successfully measured the body sizes of wild Painted Storks (*Mycteria leucocephala*) in two different biogeographic regions of India, spatially separated by 20° of latitude. We have used the wild Painted Storks as model species for measuring their morphometrics using a non-invasive technique that could easily be applied to similar species, rare, endemic, colonial, aquatic, and even those with cultural taboos. Our results satisfactorily classify and predict the sexes of the species and their biogeographic origin based on independent morphological variables using Machine Learning algorithms. The BayesNet yielded the correct classification instances (Receiver Operating Characteristic (ROC) = 0.985), outperforming all the other tested classifying algorithms. A strong relationship was observed between the local bioclimatic conditions and the morphological variations in wild Painted Storks reflecting clear eco-geographic patterns. Without this non-invasive technique, it would be almost impossible to collect morphological measurements at a large scale from live birds under field conditions. Our study is a testimony to the effectual use of the non-invasive digital method for in-situ measurements from free-living wild species in the field, assuming significance, especially from climate change perspectives, biology, ecology, and conservation.

## Introduction

Morphological measurements of species are critical for understanding certain fundamental aspects of their biology, especially the developmental aspects of life history, sexual dimorphism, the relationship between body sizes and reproductive success or fitness, and overall health^1–4^. For morphometric measurements by the conventional method, physically capturing and handling organisms are indispensable. Free-living animals demand sedation or physical restraint, often leading to stress or trauma, altering their original behaviour, or even post-release death. Sedation helps make measurements, but often under various constraints, such as within a short time, the researchers must complete all the measurements, requiring highly skilled veterinarians. Moreover, sample size constraints are unavoidable and are laborious, cumbersome, and time-consuming. On the contrary, photogrammetry measures animal sizes from their photographs avoiding the animal's cumbersome capturing, physical handling, and associated errors. Moreover, the non-invasive method can generate accurate morphometric measurements at a comparatively low cost^5^.

Spatial variation in the morphological characteristic is vital for survival; notably, endotherms (birds and mammals) face substantial challenges in colder environments to maintain a constant body temperature and minimize energetic costs^6,7^. Bergmann’s^8^ and Allen’s^9^ rules state that the organism in colder environments and those in higher latitudes and altitudes tend to be larger with smaller appendages to minimize heat loss. Studies on birds reveal considerable evidence for both the rules directly correlated with morphology and temperature^2,10^. Temperature variation due to latitudinal differences has been a potential driver of morphological adaptation^6^, signals to quantify effects of climate changes, and consequent variations in climatic features, as shown by Raju et al.^11^ in the case of Indian districts. It is likely that bio-climatic variations, on a long-term basis, would have important implications on species sizes and morphological features^12^. Morphological differences due to sexual size dimorphism (SSD) in birds were reported^13–15^, and measurements for such works have been collected from captive specimens, which are increasingly becoming difficult in the case of many wild species facing various types of existential pressures.

We have used the wild Painted Storks (*Mycteria leucocephala*), a model species for measuring morphometrics using non-invasive techniques that could easily be applied to similar species of rare, endemic, colonial, aquatic, and even species with cultural taboos. The Painted Stork has a widespread distribution in different biogeographic regions of India, at the same time showing site fidelity to their traditional nesting areas. The Painted Stork is a colorful, monomorphic, large wading bird, widely distributed in the Indian subcontinent^16^ and parts of Asia^17^. They congregate in traditional nesting colonies or heronries as distinct sub-populations during breeding seasons. This species is breeding for two seasons spread across different biogeographic zones in India. However, the main hurdle in studying the morphology of such a large bird is the restrictive laws and the species being listed as Near-Threatened in India^18^, so capturing is often discouraged, apart from several other practical difficulties. We could overcome such hurdles by adopting a non-invasive/non-destructive method to explore their external size/shape variations through digital photographs. We also applied a technique^5^ to extract the clues of eco-geographic variations and sexual dimorphism among different populations of the same species. Further, we have explored the morphological datasets on the Storks to classify and predict the sexes and regions of their origin using Machine Learning (ML) algorithms, an artificial intelligence (AI) technique.

## Results

**Figure 1:**
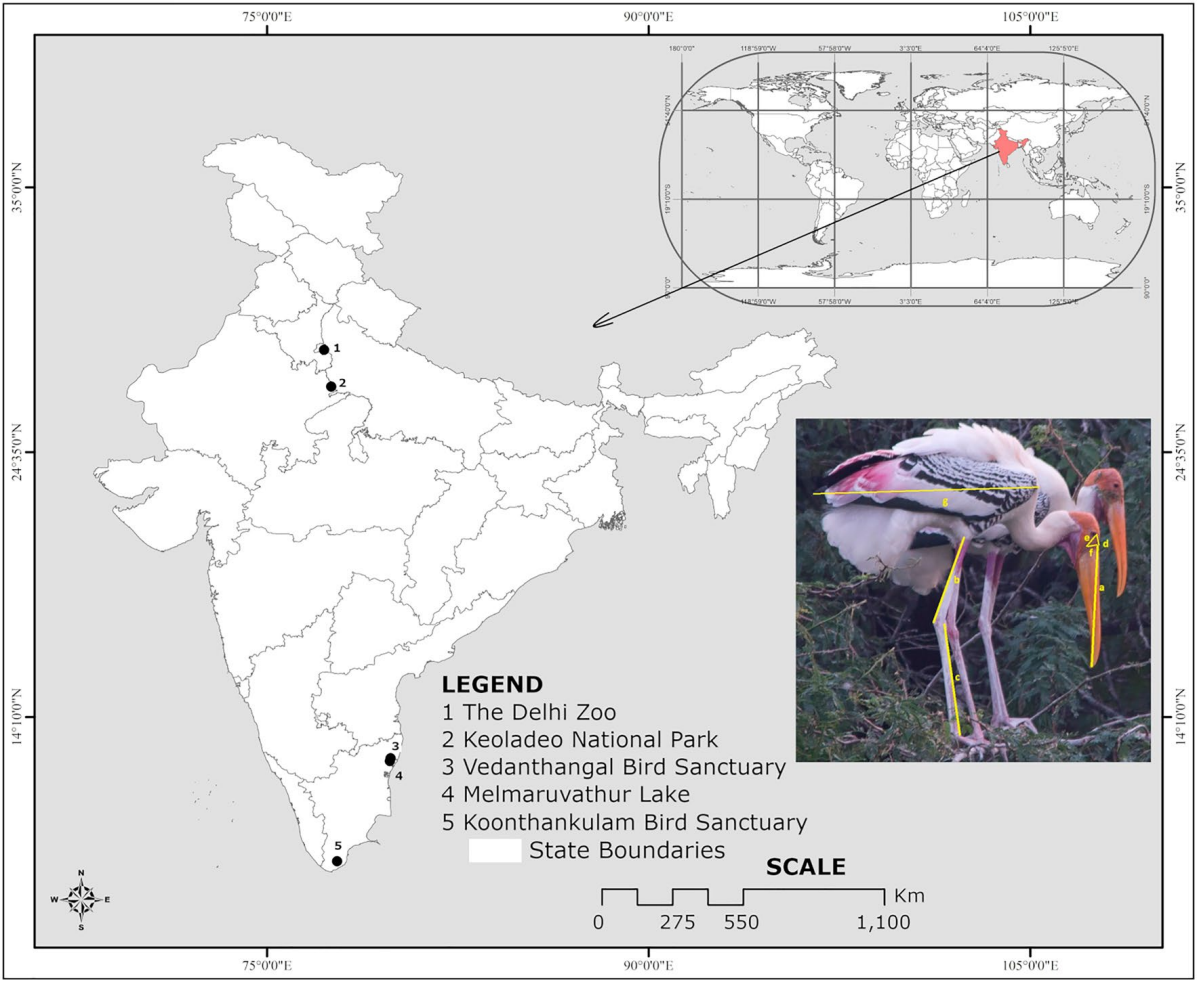
Locations of the selected nesting colonies of the Painted Storks in the northern region of India are marked as 1 & 2, while areas in southern India are marked as 3, 4 & 5: Inset shows the digital image of Painted Storks. The map was prepared by open source Q GIS 3.12 București & GIMP 2.10.32.

The breeding colonies of Painted Storks from the north and south India are separated by nearly 20° of latitude; hence, eco-geographical and bio-climatic variations are expected to be found in them (Fig. 1).

**Figure 2:**
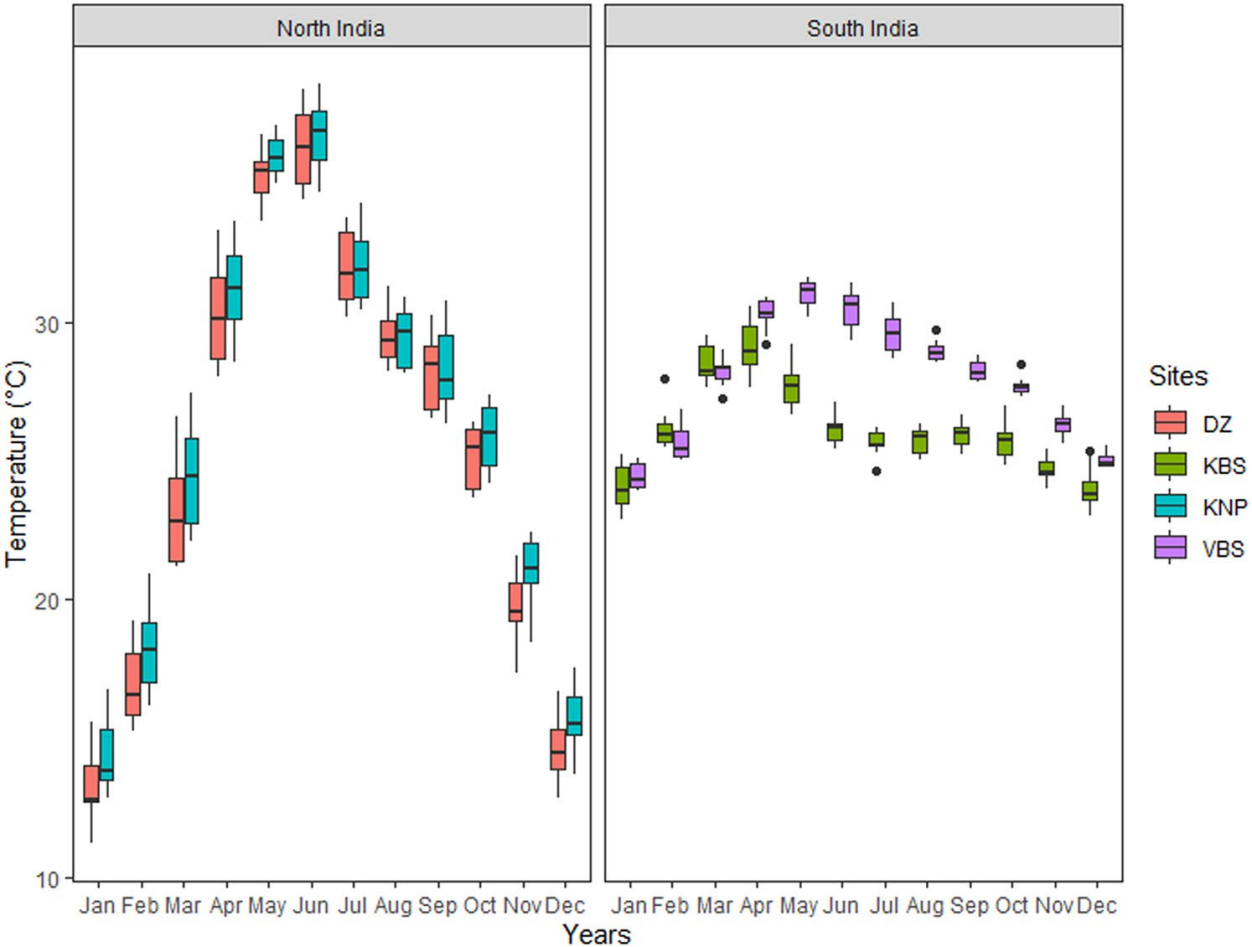
Monthly variation of Temperature (at 2 m from ground level) at the study sites during 2010–2020 years (*DZ* Delhi Zoo, *KBS* Koonthankulam Bird Sanctuary, *KNP* Keoladeo National Park, *VBS* Vedanthangal Bird Sanctuary).

The air temperature of the selected colonies in north India is slightly higher (Mean = 27.73 °C, SD ± 7.5) than in South India (Mean = 25.06 °C, SD ± 2.2) with a large effect size (Cohen’s d estimate: − 6.32, with 95% Confidence Interval − 6.613 to − 6.022). A two-way ANOVA showed that the sites did have a statistically significant temperature difference (*p* = 2e^−16^). Even the months also showed a statistically significant temperature variation (*p* = 8.62e^−09^) (Fig. 2). The interactions between sites (North and Southern regions) and months of the year (F _(__11, 240__)_, F = 244.6, *p* = 2e^−16^) are also statistically significant.

### Morphometric variations in Storks from the north and south India

**Figure 3:**
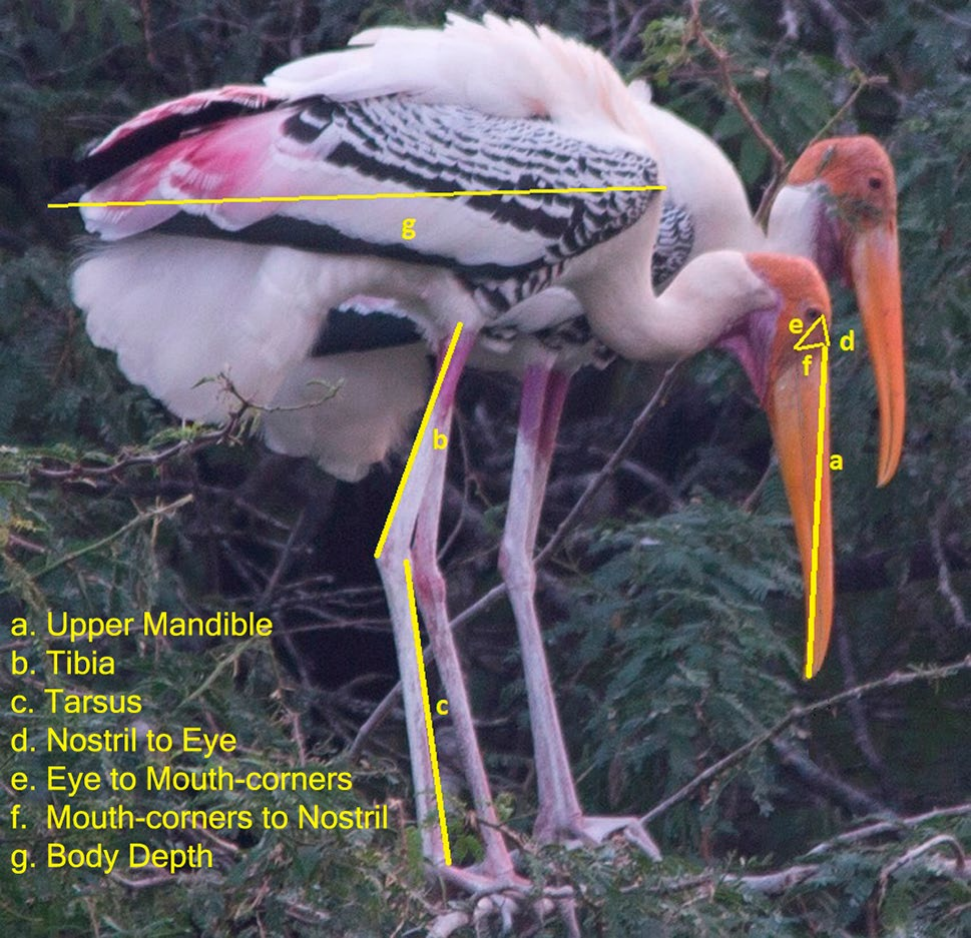
The digital image of Painted Storks and their selected morphological variables are shown on the bottom left. The length of the Upper Mandible marked in yellow as ‘a’ is 21 cm; details of other morphological variables are explained in the text (“Measurements of the morphological variables”).

Following the criteria discussed in the methods, digital images of the 148 individuals of Painted Storks, 99 (51 males and 48 females) from North India (NI) and 49 (24 males and 25 females) from South India (SI), were included for the analysis. Morphological characters, viz., the Upper mandible (Bill length), distances among nostril, eye, and mouth corners, left and right sides of tibia and tarsus, and body depth of the Storks from the north and south Indian populations were estimated (Fig. 3; Table 1). Based on two-sample *t*-tests, significant differences in most morphological measurements (bill lengths, nostril-eye distance, mouth corners to nostril distance, tibia, and tarsus) between the sexes indicated substantial differences in sexual dimorphism in both Indian populations (Table 1).

**Table 1:** Summary of the morphological variables of Painted Storks measured in units (cm) from two (North and South) regions of India, represented with the mean (± SE) and the p-values indicate the statistical differences between males and females.

Morphometric variables	Northern region			Southern region		
	Male (N = 51)	Female (N = 48)	Two sample *t*-test	Male (N = 24)	Female (N = 25)	Two sample *t*-test
Upper mandible	20.9514 ± 0.2649	17.936 ± 0.2591	*t* (97) = − 8.1252;* p* = 1.454e^−12^	20.0943 ± 0.4935	17.9776 ± 0.4596	*t* (47) = − 3.1419;* p* = 0.002904
Nostril to eye	2.6758 ± 0.0421	2.2013 ± 0.0584	*t* (97) = − 6.6555;* p* = 1.688^–09^	21.835 ± 0.0482	2.2381 ± 0.0682	*t* (47) = 0.64988;* p* = 0.5189
Eye to mouth-corners	2.4626 ± 0.0784	2.3509 ± 0.0584	*t* (97) = − 1.1321;* p* = 0.2604	2.9241 ± 0.1172	2.5693 ± 0.0655	*t* (47) = − 2.6727;* p* = 0.01031
Mouth-corners to nostril	2.4115 ± 0.0772	2.133 ± 0.0747	*t* (97) = − 2.5882;* p* = 0.01113	2.9512 ± 0.1442	2.1811 ± 0.0714	*t* (47) = − 4.8473;* p* = 1.407e^−5^
Left tibia	15.6174 ± 0.2824	14.8919 ± 0.1655	*t* (97) = − 2.1829; *p* = 0.03145	13.8858 ± 0.4085	13.5159 ± 0.2514	*t* (47) = − 0.77805;* p* = 0.4404
Right tibia	16.1323 ± 0.3105	14.4452 ± 0.2859	*t* (97) = − 3.9831; *p* = 0.00013	14.0093 ± 0.3179	13.9982 ± 0.3512	*t* (47) = − 0.02329;* p* = 0.9815
Left tarsus	18.7838 ± 0.3776	17.7437 ± 0.2616	*t* (97) = − 2.238; *p* = 0.02751	19.5122 ± 0.3770	17.4792 ± 0.3647	*t* (47) = − 3.8769;* p* = 0.0003274
Right tarsus	20.0439 ± 0.2242	17.5886 ± 0.1423	*t* (97) = − 9.1204;* p* = 1.063e^−14^	19.7042 ± 0.3549	17.9714 ± 0.4458	*t* (47) = − 3.0255;* p* = 0.004017
Body depth	41.8593 ± 0.4264	41.3183 ± 0.5889	*t* (97) = − 0.75069;* p* = 0.4547	43.2083 ± 0.7167	41.1454 ± 0.6687	*t* (47) = − 2.1066;* p* = 0.0405

### Regional and sexual variation in the body size

**Figure 4:**
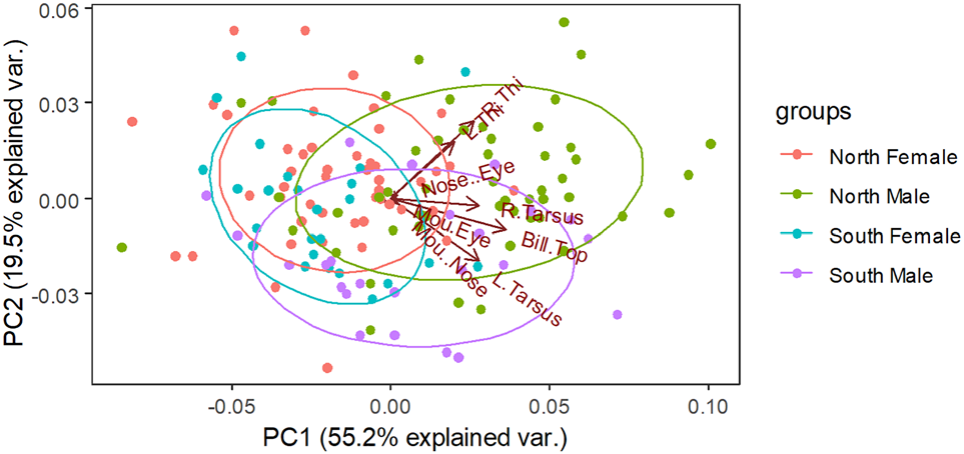
The principal component plot represents the multivariate dataset of the morphological variables of Painted Stork; the first principal component (PC_1_) is plotted along the x-axis, and the second principal component (PC_2_) along the y-axis. *NF* North Female, *NM* North Male, *SF* South Female, *SM* South Male.

Through the Principal Component Analysis (PCA), the morphological variables, a multivariate set, revealed that the first principal component (PC_1_) accounts for 52% of the total variation, with positive coefficients for all the variables representing the general body size. The second component (PC_2_) that accounts for 19.2% of the variation contrasts with two variables, viz., tibia and tarsus, the measures of the right and left sides of the tibia and tarsus as metrics of the leg morphology (Fig. 4). We noticed a medium effect size between the PC_1_ scores (body size) and the sex (Cohens d: − 4.15968) and a small effect size between PC_2_ scores (body shape) and the sex (Cohens d = 2.629459). Therefore, nested ANOVA of the PC_1_ showed minor variation between regions (F_1,2_ = 0.628, *p* = 0.429) and significant variation in sexes that nested within the regions (F_1,2_ = 18.49, *p* = 7.11e^−08^). Similarly, the nested ANOVA of the PC_2_ scores indicated an apparent sexual dimorphism with a substantial variation in the leg sizes of the Painted Stork between the regions (F_1,2_ = 6.17, *p* = 0.01) and between the sexes that nested within the regions (F_1,2_ = 8.52, *p* = 0.0003). Clear evidence of the operation of eco-geographic rules between the regions rendering the body size variation and sexual dimorphism in the species, particularly in males, were observed (Fig. 4). It is probably why the Nested-ANOVA predicted the regional differences in the body sizes (Table 2).

**Table 2:** The principal component scores as the dependent variables, while the regions (north and south) and sexes from both populations (coded as NM, NF, SM & SF) as factors in the nested-ANOVA. The population representing sexes is nested within the region.

S. no	Dependent variable	Factors	Df	Sum of squares	Mean square	F value	p value
1	PC_1_ scores	Region	1	0.00079	0.000790	0.628	0.429
		Region: factors ( populations including sexes)	2	0.04648	0.023238	18.492	7.11e^−08^
		Residuals	144	0.18095	0.001257		
2	PC_2_ scores	Region	1	0.00557	0.005571	6.175	0.014098
		Region: factors( populations including sexes)	2	0.01538	0.007688	8.522	0.000318
		Residuals	144	0.12991	0.000902		

### Identifying Stork populations using Machine Learning (ML) algorithms

The multinomial regression categorized Painted Stork populations as statistically distinct from the reference (NF) in more than five morphological variables (Table 3), enabling us to predict the sex of the individuals and the region to which it belongs. The nine independent morphological variables, drawn from digital images, were sorted with sex and regions coded as nominal variables.

**Table 3:** Multinomial logistic regression model shows the morphological variables associated with different populations of Painted Storks among sexes and regions, with the north female (NF) Painted Stork as the reference category.

Morphometric variables	Dependent variable					
	NM		SF		SM	
	(logit 1)		(logit 2)		(logit 3)	
	Coefficient	± SE	Coefficient	± SE	Coefficient	± SE
Upper mandible	56.509***	19.999	− 6.079	18.433	15.282	25.076
Nostril to eye	331.546***	97.247	− 10.466	80.636	− 109.723	113.952
Eye to mouth-corners	− 98.386	81.960	126.742*	73.415	198.809**	86.971
Mouth-corners to nostril	114.162*	68.273	− 36.399	59.031	195.077**	80.197
Left tibia	− 23.584	26.276	− 97.447***	26.431	− 98.051***	34.742
Right tibia	− 8.172	23.18	11.841	19.041	− 14.513	32.119
Left tarsus	− 35.881**	18.157	− 5.237	16.9	38.518	28.973
Right tarsus	102.353***	28.623	45.275*	23.731	68.441**	30.823
Body depth	− 11.700	8.725	− 1.693	8.094	18.107	11.609
Constant	− 22.306***	5.544	4.133	5.85	− 22.500***	7.225
Akaike inf. crit	253.573		253.573		253.573	

*p < 0.1; **p < 0.05; ***p < 0.01.

**Figure 5:**
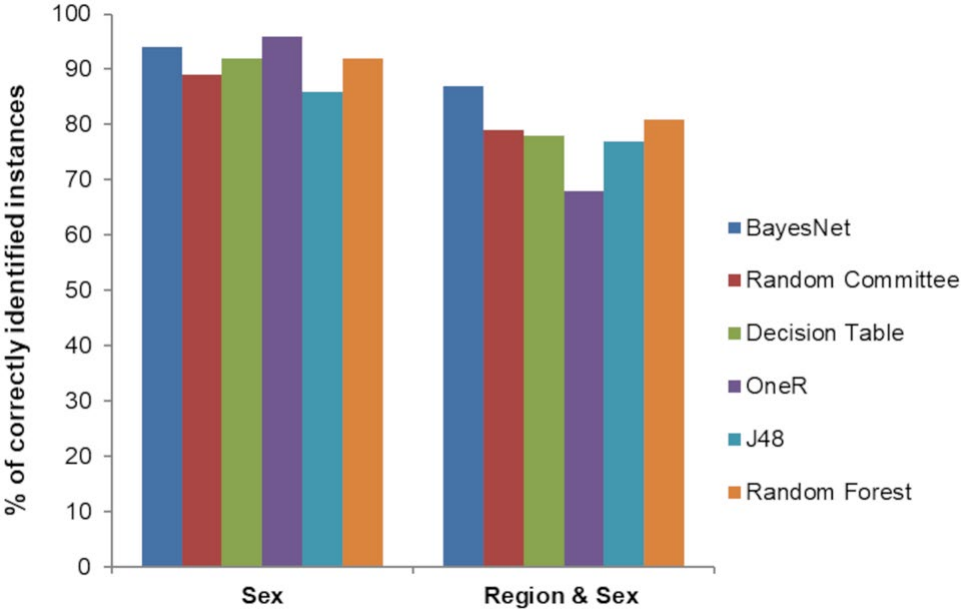
Top Machine Learning algorithms with the percentage of correctly classified instances of Painted Storks with Sex as classifier and Region & Sex as another classifier.

With the WEKA classifiers, we classified and predicted the sex of the Storks from the independent morphological variables drawn from digital images. Compared with other ML algorithms, the rules OneR got the highest correctly classified instances. The BayesNet yielded the best values (Precision = 0.940, Recall = 0.939, F-Measure = 0.939, ROC Area = 0.985), with a significant level (Confidence: 0.05, two-tailed Paired-T-Tester), which outperformed all the other classifying algorithms (Table Supplement 1; Fig. 5). Keeping the sex and region as nominal variables (coded as NM, NF, SM, and SF), we again classified and predicted the Storks with independent morphological variables. Among all the classifiers the BayesNet yielded the highest correctly classified instances (Precision = 0.874, Recall = 0.872, F-Measure = 0.872, ROC Area = 0.97), with a significant level (Confidence: 0.05, two-tailed Paired-T-Tester) outperforming all the other classifying algorithms (Table Supplementary 2; Fig. 5).

## Discussion

The non-invasive study shows the variation in morphometrics between two regional populations of wild birds and between the sexes. It also demonstrates the suitability of the non-invasive digital methods by Mahendiran et al.^5^, a trigonometric approach for in-situ estimation of wild species morphometrics, using Painted Stork as a model. Compared to other methods^15^, this method gives better (mm level) accuracy in estimating the morphometric measurements. Without the in-situ method^5^, it would be almost impractical to collect morphological measurements at a large scale from wild live and free-ranging species under field conditions, especially if the species are listed in the Near-Threatened category, as in the case of our model species, the Painted Stork^18^. Capturing such species (as is the case for many more wild species) for morphometric measurements is often discouraged. Further, the individuals would be under tremendous stress while being measured^15,19^, with severe consequences. Our non-invasive method is often a win–win strategy for taking large-scale morphological measurements while ensuring a genuine animal welfare approach.

The sexes were distinct with the morphological variables, particularly in the bill and leg measurements, indicating sexual dimorphism in the Painted Storks, an outwardly monomorphic species. With the help of PC_1_ and PC_2_ scores, it is possible to understand the differences in sexual dimorphism (Table 2). Mate preference is attributed to SSD, where the females prefer larger males primarily because they are more successful in obtaining territories^20,21^. Robust sexual selection, with a positive assortative mating for body size in our model species and several others^15,22^ have been reported. The basal metabolic rate, success in mating, food resource availability, and competition are essential cofactors for the body size variations in birds^23–25^. In addition, the sex ratio, the social structure, and socio-ecological situations (including courtship repertoire, mating patterns, or sexual selection) could directly influence the sexual selection in the Painted Storks. Despite the sexual dimorphism index of 85% in bill length and as close to 95% with tibia and tarsus, it is difficult to find the sexual differences in the birds by observers through their naked eyes. In certain species, contemporary global issues such as climate change are reported to alter sexual selection and avian phenology^26^. Studying sexual selection, its modifying factors, and its possible effects concerning adaptations for impending global warming and its consequences requires quantifying species' body sizes based on either single or multivariate traits. However, depending on any single morphological feature, as a proxy for body size, without considering that those specific features that may be under different selection pressures would be inexact^27–29^. For example, bills for feeding and heat dissipation, wings for flying, tarsus for walking, foraging, and wading, and such specific functions would be under strong selection pressures; thus, considering measurements based on single features would be imprecise. Selecting any single morphological feature may not reveal patterns of sexual selection or reflect the overall variation in body size following Eco-geographic rules. Therefore this study estimated the general body sizes from the scores (PC_1_ & PC_2_) derived from the multivariate morphological variables of the non-feathered leg, bill, and head regions that probably undergo the same or closely related selection pressures and reflect the overall growth of their body sizes. The morphometric traits loaded on PC_1_ and PC_2_ are good descriptors of general body size and shape, respectively (Fig. 4).

We noted significant differences between the northern and southern regions (Fig. 2) in the bioclimatic variables because of the existing latitudinal differences. A humid subtropical climate predominates in the north, with hot summer and cold winter temperatures as low as 0 °C. In contrast, in southern India, spread over the south of the Tropic of Cancer, a hot semi-arid, drought-prone climate predominates and tends to have unreliable rainfall due to sporadic lateness or failure of the southwest monsoon, according to the Köppen system^30^. The Painted Storks breed with the closure of the southwest monsoon (Aug-Feb) in Semi-arid (4A-Punjab plains and 4B-Gujarat Rajputana and Gangetic plains (7A &7B)) of North India, while in the Deccan Peninsula, Deccan south (6E) and West Coast (8A) it starts with the onset of the northeast monsoon (Nov–Apr)^16,31^. This disparity in the species' breeding seasons illustrates the biogeographical variations and environmental influences. We observed Painted Storks in distinct pockets of population restricted only to traditional nesting colonies or heronry, at most showing local movement to nearby local heronries. As there is no record of long-range migration of the Painted Storks across the sub-continent, the spatial distance between the two biogeographic zones probably acts as a barrier to genetic exchange^32^.

Moreover, a combination of climatic factors, particularly temperature, could influence bird body size, reflected subtly in our Nested ANOVA results. Nevertheless, Bergmann's and Allen's rules may not precisely match the present study regions because neither of them experiences continuous cold conditions throughout the year. The northern sites of our studies reach high peaks in summer temperature and very low in winter. However, this wide temperature variation could challenge the species' thermal optima for its subpopulations, expanding their tolerance thresholds. The northern region shows the high variance in temperature and other bio-climatic and environmental variables, supporting subtle eco-geographic trends. In contrast, the southern region does not experience drastic variations, especially in temperature. A combination of climatic factors, such as the temperature and moisture, could influence the body size variation in birds where the smaller body size dominates in hot and humid conditions, while the larger size in colder and drier conditions^2,10^. At global and local scales, atmospheric variables, such as wind pattern, temperature, precipitation, ozone, climate oscillations^33^, and even teleconnection patterns influence a range of biological and ecological facets of birds such as breeding, fledging, breeding success, molting, and migration phenology^33^.

Sexual dimorphism indicates the strong sexual selection dominating in the Painted Stork than the eco-geographic variations, as evident from non-significant interactions in the nested-ANOVA. It shows clear trade-offs between sexual dimorphism and eco-geographic variations, possibly for the limited plasticity in morphology of the species (Table 2). Despite limited plasticity in the body size of birds, climate change probably leads to a reduction in the body size in non-migratory understory bird communities^34^. The long-range migratory birds^35^ possibly affect avian physiology, phenology, and other associated activities^33^.

As we identified the sexes of Painted Storks based on their orientation during copulations, and further, we confirmed them based on the morphological measurements derived from the digital photographs taken during those acts. Several studies^36,37^ have demonstrated that sexing animals could be done based on the ratio of body parts. Our results using multinomial logistic regression is a typical example of classifying and predicting significant differences in more than five variables based on the region (of their origin) and sexes. At the same time, we cannot rule out the possibility of extra-pair and same-sex pseudo-copulations because the negative sign in the multinomial regression coefficient indicates an increase in the predictor variable (female body size) with the decrease in the response variable (male body size). Similarly, the 7% difference between the right and left tarsus of northern male storks is an interesting puzzle in fluctuating asymmetry. The answers to the puzzle may be in behaviour of the birds, particularly with the mating pattern. For example, assortative mating (larger male paring with larger female) has been reported in Painted Stork^15^. Our data on tibia and tarsus shows a negative trend, meaning as female leg length increases, the male leg length decreases in the northern population of the Painted Stork. It might be due to pseudo copulations and extra-pair copulation (probably with helpers or young individuals), and signals may influence such behaviour as social causes for the mating pattern. Therefore, these signals may indicate such behaviors as social causes.

Though sexual differentiation using the naked eye is very subtle, we first showed that using the Painted Stork morphometric variables to identify and differentiate the sex and classify them under different regions of their origin using ML algorithms. As the sexual differences were substantial compared to regional differences, the accuracy and areas under the curve (ROC area) showed adequate differences in ML algorithms (Fig. 5). We successfully showed the non-invasive sexing of birds under field condition that assumes research and conservational importance. Though we had collected morphometric measurements from the right and left sides of the birds, we restricted the analysis only to check for the variations between sexes and regions. There is excellent space left to explore questions relating to fluctuating asymmetry using this technique and sex/individual recognition through deep learning frameworks with cloud-based GPU/TPU, but it is beyond the scope of this work.

## Materials and methods

### Study area

We conducted field studies in both regions from August to March, each year from 2012 to 2016. In north India, we selected the two traditional breeding colonies of the Painted Storks, viz., the Delhi Zoo (28° 36′ N 77° 14′ E) and Keoladeo National Park (KNP) (27° 17′ N 77° 52′ E), Bharatpur, Rajasthan (Fig. 1). In the Delhi Zoo, close to the river Yamuna, the Painted Storks nest in the traditional heronries with other colonial nesters, Little Cormorant, Indian Cormorant, Black-headed Ibis, and Night Heron^38^. The KNP, a Ramsar site spread over 29 km^2^, situated at the confluence of the rivers Gambhir and Banganga on the western edge of the Gangetic basin, supports diverse fauna, flora, and a mosaic of habitats, wetlands, woodlands, scrub forests, grasslands, and heronries^39^. In 2013, we recorded 680 adults and 310 nests in the Delhi Zoo and 1584 adults and 430 nests of Painted Storks in the KNP.

We selected the Vedanthangal Bird Sanctuary (VBS), the nesting colonies at Melmaruvathur Lake, and Koonthankulam Bird Sanctuary (KBS). The KBS & VBS are the newly declared Ramsar sites in Tamil Nadu, south India. The VBS (12° 32′ 02″ N and 79° 52′ 29″ E) is a 40.3-hectare community reserve effectively protected by the state Forest Department, Tamil Nadu, and Vedanthangal villagers^40^. It is the oldest breeding waterbird reserve in south India, located 85 km southwest of Chennai. More than 40 species of waterbirds, both residents and migrants, live here. Along with the other 17 heronry species, the Painted Storks build nests every year from November to April during its breeding season. The Painted Stork nesting colonies at Melmaruvathur Lake (12° 25′ 53″ N and 79° 49′ 36″ E) are about 20 km away from the VBS. Here, the Painted Storks build nests at 1.8–5 m above the water level, on trees of *Acacia nilotica* and *Barringtonia acutangula* on mounds surrounded by water^41^. In 2012, we recorded a total of 3185 nests in the VBS, with a maximum number of nests belonging to Spot-billed Pelican (1050 nests) followed by Painted Stork (550 nests), Asian Open-bill (770 nests), and others.

Birds have been breeding in Melmaruvathur Lake since 2013, and we counted 80 nests of Spot-billed pelican, 45 nests of Oriental White Ibis, and 56 nests of Painted Stork during the winter of the year 2014. The Lake is spread over 0.19 km^2^ with islets (mounds) with four clusters of *Acacia nilotica* and *Barringtonia acutangula* trees. Rainwater and domestic sewage from the neighboring residential complex are the primary water source, and recreational boating attracts a large crowd visiting the Melmaruvathur temple^41^. KBS (8° 29′ 44″ N and 77° 45′ 30″ E) is about a 1.3 km^2^ protected area, declared a bird sanctuary in 1994 and an Important Bird Area^40^. It comprises Koonthankulam and Kadankulam irrigation tanks actively protected and managed by the local community. We noticed the frequent failures of breeding events due to water shortages related to monsoon failures in VBS and KBS. In 2015, we also observed Painted Storks' breeding failure across northern India for unknown reasons; therefore, data could not be collected for those periods.

### Bioclimatic variables

We obtained the bioclimatic variable, particularly temperature at 2 m height for all the four study sites, from the National Aeronautics and Space Administration (NASA) Langley Research Center (LaRC) Prediction of Worldwide Energy Resource (POWER) Project funded through the NASA Earth Science/Applied Science Program. The monthly average data from 2010 to 2020 was downloaded from the POWER Project's Hourly 2.0.0 version on 2022/01/04.

### Digital images of Painted Storks collected under field conditions

Using Binoculars (Olympus 10X50), Digital Cameras (Canon 5D Mark III and Sony handy-cam), we monitored and recorded all active nests with juveniles and adult Painted Storks twice a week. The nests were on trees, 3–7 m in height, and chicks and adults were visible, which aided the photography. Nests were numbered for our records by taking note of tree branching patterns, the nest's position on the tree, and other local identification marks. Numbering the nests helped us identify the individuals associated with a given nest and avoided re-recording the same individual (pseudoreplication). Storks show site fidelity^42,43^, and hence we assumed the same breeding pairs occupied the same nesting site.

During the initial months of the breeding seasons, pairing and copulations of the breeding pairs could be readily noticeable. We took consecutive photographs when they were copulating at the nest. After disengagement following the copulation, the birds (male and female) standing side by side at the nest were also photographed. The first author noted all the relevant spatial orientations of males and females during each copulation event in the field notes. Thus nearly 100 copulations involving different individuals of the Painted Storks pair were photographed. To minimize measurement errors, we selected for further analysis only the images of males and females standing parallel and close to each other, perpendicular to the camera. Since we used the digital images of the free-living Storks, we did not have the freedom to choose all morphological features resulting in some missing values. Therefore, we selected a hundred and forty-eight individuals for the analysis from nearly 1500 localized adults. The technique has an efficiency of less than 10% of the population, more efficient than the traditional capture, measure, and release of individuals. Though many individuals were recorded, only a few were subjected to the analyses. Moreover from the digital images, not all the morphological characters of the individuals were measured. The birds' orientation towards the camera assumes importance because the correct direction ensures maximum exposure of body parts in the picture. In many pictures, correct orientation was missing as the birds were behind other individuals or branches of the trees or leaves. Therefore, selecting the right digital image becomes crucial. Keeping all the above criteria, we filtered images that were later included in the analysis.

### Calibrations of subject-distance using Exif Metadata

We extracted the EXIF metadata from each JPEG image of Painted Stork. EXIF metadata includes the filename, type, date, and time of the image captured, image width and height in pixels, camera model, lens information, field of view, focal length, and subject-distance. The subject-distance (Painted Stork distance from the camera) being a critical variable and its Exif metadata were standardized with the following equation.

1 $${\text{Subject{-}distance}} = 0.7864 \times {\text{(EXIF subject{-}distance)}}^{{1.0301}}$$

Using the Eq. (1) derived from an earlier study^5^, we regressed actual subject-distance with the Exif subject-distance from the images. Then multiplying with the field of view, available as Exif metadata (angle of view) with standardized subject-distance (Eq. 1), the total image size (length and width) in metric units was estimated. We excluded the cropped or manipulated images because Image (size) estimation is possible only for the images coming straight from the camera with EXIF tags. The methodological details for calibration and estimation of in-situ measurements of the morphological variables are given in Mahendiran et al.^5^.

### Measurements of the morphological variables

We created a TPS file for JPEG images of Painted Storks with the TPSUtility Program^44^. Using the TPS file in the TPSDig (v. 2.17) program^44^, we measured the selected characters (morphological variables) in pixels. Later, it was used along with the total image size to estimate the size of the specific morphological features in metric units, following Mahendiran et al.^5^. Ten different morphological variables were measured: Bill length (upper and lower mandible), tibia & tarsus length of both legs, distances among the ear, nostril and corners of the mouth, and body length. We estimated the dimensions of the rigid body parts, viz., bill length, tibia, and tarsus using the given methodology^13,15,21^. Bill length is the distance from the tip of the upper mandible to the beginning of skin corners near nostrils, the proximal end of the beak (marked as ‘a’ in Fig. 3); Tibia length is the distance from the joint of the tibia-tarsus to the feathers (marked as ‘b’ in Fig. 3); Tarsus length is the distance between the tibia-tarsus joint and foot (marked as ‘c’ in Fig. 3). We took measurements of each individual's right and left legs and other characters, viz., inter-distances among the nostril, corner of the eye, corner of the mouth on each side (marked as ‘d’, ‘e’, ‘f’ in Fig. 3). Body depth is the distance from the base of the neck near the breast to the tip of the tail (marked as ‘g’ in Fig. 3).

### Data analysis

We performed the statistical analysis in R^45^, primarily through the nlme, ggbiplot, nnet, tidyverse, devtools packages. We did not have the freedom to measure a few morphological variables due to the problems mentioned above, which led to missing values in the datasets. We filled the missing values with the impute function using the R Core team^45^ through mice & VIM packages. When the missing values are high in numbers, we discard the data rather than use the impute function. Since almost about 70% of the lower mandible values were missing, we discarded them and ended up having only nine morphological variables in the final analysis. Moreover, the lower mandible is movable, with the mouth being open and closed, producing a considerable variation in measurements.

We designed the matrix (Individuals × Region × Sex) representing the intraspecific variations concerning the region and sexes of Painted Storks^46^. The individuals are in rows (R), their region in column (C_1_), and sex in column (C_2_). We considered the regional variations as a sequence of the latitudinal gradient of the study sites. The values of the individuals (R) were the selected morphological variables. This matrix helped us investigate the critical questions relating to eco-geographic variations and sexual dimorphism.

To determine whether temperature varied between study sites, we conducted a two-way ANOVA to analyse the effect of study sites (between North India (DZ & KNP) and South India (VBS & KBS)) and months of the year on the temperature at 2 m. For each character, Dimorphism Index (DI) was calculated as a mean value of female divided by the mean male, multiplied by 100, following the method of Urfi and Kalam^15^. We estimated the general body size of Painted Storks from the selected morphological variables through Principal Component Analysis (PCA) and tested hypotheses on Eco-geographic variations (Bergmann's or Allen's rules)^2,47^ and the sexual dimorphism^15,48^. The dimension reduction through PCA was carried out after the imputation as there were a few missing values. Body depth was omitted only for the principal component analysis due to many missing values. However, the values of all the characters are presented in the summary statistics in Table 1. The first principal component is characterized as a measure of size, and subsequent components describe various aspects of shape; therefore, it is considered a measure of general body size^15,48,49^. The PC_1_ indicated the body size variation, and PC_2_ revealed leg length variation (tibia and tarsus). We used nested ANOVA to test their body size variation between regions and sexes. The sexes nested within the region explained the eco-geographic rules and sexual selection patterns.

Using a multinomial logistic regression model, we compared the Painted Storks’ northern male (NM), southern male (SM), and female (SF) with the reference category, northern female (NF). Then, we classified the data through multinomial log-linear and feed-forward neural network models. We predicted the Painted Stork's region and sex using the Machine Learning (ML) algorithms through open-source software Waikato Environment for Knowledge Analysis (WEKA.3.9.5) implemented in Java^50^. WEKA has standard Machine learning/data-mining algorithms with pre-processing tools generating insightful knowledge from the Painted Storks' morphological data.

Using the R and Python interfaces, we used different ML software frameworks, libraries, and computer programs, viz., TensorFlow and Keras, and extensively explored the WEKA workbench environment to predict the sex and region of the Painted Stork. We used the k-fold cross-validation (k = 10) to avoid overlapping test sets, including splitting the data into k subsets of equal size, using each subset for testing and the remainder for training. We analyzed using the WEKA on a Lenovo ThinkPad P53s Mobile Workstation with the 8th Gen Intel® Core i7 @ 1.80 GHz processor, 48 GB DDR4 Memory, NVIDIA® Quadro® P520 with 2 GB GDDR5 Graphics. The performance criteria for all the eight models were assessed by using the Precision (TP/(TP + FP)), Recall (TP/(TP + FN)), Area under Curve (AUC) = (Sensitivity + Specificity)/2, Accuracy = (TP + TN)/(TP + TN + FP + FN), where TP, TN, FN and FP are the acronyms of true positive, true negative, false negative and false positive, respectively. We used the WEKA experimenter environment to test the statistical significance of the selected Machine Learning algorithms. We performed the Paired T-tester based on the number of correctly classified instances and areas under the curve.

## Supplementary Information

Supplementary Tables.

## Data Availability

All relevant information of this experiment, viz., the selected morphological variables extracted from the digital images of Painted Storks, are deposited in a public repository (Mahendiran & Parthiban, 2021). https://datadryad.org/stash/share/fgLFJWYtjeYYZZ49VYCK58rjejifQ3f78h8NneKy4OA.
