# Supplementary material for: Signals of local bioclimate-driven ecomorphological changes in wild birds
Source: Sci Rep. 2022 Sep 27;12:15815. doi: 10.1038/s41598-022-20041-w (PMC9515120; doi:10.1038/s41598-022-20041-w)
Supplement: Supplementary file 1 — Supplementary Tables. [file 41598_2022_20041_MOESM1_ESM.pdf]

Title: Signals of local bioclimate-driven ecomorphological changes in wild birds

Authors: Mylswamy Mahendiran [1]*, Mylswamy Parthiban [2, 3], and Parappurath Abdul Azeez [1, 4]

Institute affiliation:

[1, 4] Sálim Ali Centre for Ornithology and Natural History, Anaikatty, Coimbatore - 641 108, Tamil Nadu, India

Email[1]: mahenrose@gmail.com (* Corresponding author)

Mylswamy Mahendiran http://orcid.org/0000-0002-9938-5742

[2]Agricultural College and Research Institute, Tamil Nadu Agricultural University, Coimbatore - 641 003, Tamil Nadu, India

[3]Post Graduate & Research Department of Computer Science, Government Arts College (Autonomous), Affiliated to Bharathiar University, Coimbatore – 641 018, Tamil Nadu, India

Email[2]: mp79@tnau.ac.in

Mylswamy Parthiban http://orcid.org/0000-0001-8474-1655

[4]Department of Environmental Science & Management, Bharathidasan University, Tiruchirappalli - 620 024, Tamil Nadu, India

Email[3]: azeezpa211@gmail.com

Parappurath Abdul Azeez http://orcid.org/0000-0002-9953-6459

Table supplementary 1. Machine learning algorithms predicted the sex of the Storks from the independent morphological variables drawn from the images.

S.No	Machine Learning Algorithms	Precision	Recall	F-Measure	ROC Area
1	The BayesNet	0.94	0.939	0.939	0.985
2	meta classifier RandomCommittee	0.89	0.89	0.943	0.943
3	rules DecisionTable	0.92	0.926	0.926	0.943
4	Rules OneR	0.949	0.946	0.946	0.945
5	trees J48	0.858	0.858	0.858	0.898
6	trees RandomForest	0.92	0.919	0.919	0.952

Table supplementary 2. Machine learning algorithms predicted the sex and regions of the Storks from the independent morphological variables drawn from the images.

S.No	Machine Learning Algorithms	Precision	Recall	F-Measure	ROC Area
1	The BayesNet	0.874	0.872	0.872	0.97
2	meta classifier RandomCommittee	0.795	0.791	0.787	0.941
3	rules DecisionTable	0.789	0.784	0.78	0.914
4	rules OneR	0.674	0.682	0.66	0.772
5	trees J48	0.771	0.77	0.77	0.850
6	trees RandomForest	0.811	0.811	0.810	0.951
